# Supplementary material for: A single-parasite transcriptional atlas of Toxoplasma Gondii reveals novel control of antigen expression
Source: eLife. 2020 Feb 17;9:e54129. doi: 10.7554/eLife.54129 (PMC7180058; doi:10.7554/eLife.54129)
Supplement: Transparent reporting form [file elife-54129-transrepform.docx]

***eLife’s* transparent reporting form**

We encourage authors to provide detailed information *within their submission* to facilitate the interpretation and replication of experiments. Authors can upload supporting documentation to indicate the use of appropriate reporting guidelines for health-related research (see [EQUATOR Network](http://www.equator-network.org/ \t _blank)), life science research (see the [BioSharing Information Resource](https://biosharing.org/)), or the [ARRIVE guidelines](http://www.plosbiology.org/article/info:doi/10.1371/journal.pbio.1000412) for reporting work involving animal research. Where applicable, authors should refer to any relevant reporting standards documents in this form.

If you have any questions, please consult our Journal Policies and/or contact us: [editorial@elifesciences.org](mailto:editorial@elifesciences.org).

**Sample-size estimation**

- You should state whether an appropriate sample size was computed when the study was being designed
- You should state the statistical method of sample size computation and any required assumptions
- If no explicit power analysis was used, you should describe how you decided what sample (replicate) size (number) to use

Please outline where this information can be found within the submission (e.g., sections or figure legends), or explain why this information doesn’t apply to your submission:

**Replicates**

- You should report how often each experiment was performed

We estimated the number of single parasites to sequence based on the expected frequency of cell states that we wanted to detect from each condition. We aimed to detect ≥ 20 cells for a given cell state that may occur in cell cycle or asexual development with ≥1% frequency. This meant that we needed to sample ≥ 200 cells for each condition (with no induction or with alkaline induction). We collected and sequenced at least 300 parasites for each condition and each strain to ensure that we reach this detection limit for rare cell states from each strain/condition. The information can be found on Figure 1 and Results section “**Technical validation of single-parasite sorting and sequencing**”.

- You should include a definition of biological versus technical replication
- The data obtained should be provided and sufficient information should be provided to indicate the number of independent biological and/or technical replicates
- If you encountered any outliers, you should describe how these were handled
- Criteria for exclusion/inclusion of data should be clearly stated
- High-throughput sequence data should be uploaded before submission, with a private link for reviewers provided (these are available from both GEO and ArrayExpress)

Please outline where this information can be found within the submission (e.g., sections or figure legends), or explain why this information doesn’t apply to your submission:

**Statistical reporting**

Our definition of biological replication is an independent measurement that is performed on a sample that is independently cultured. Our definition of technical replication is an independent measurement performed on the same sample. E.g. each single cell RNA-seq data point is a technical replicate from the same sample. For our scRNA-seq measurements, the number of technical replicates is equal to the number of single-cells that we have sequenced in total. For both type I RH and type II ME49 datasets, our results are derived from a single biological replicate. While for our type II Pru dataset, our results are derived from two biological replicates. For our AP2IX-1 transfection experiment detailed in the Results section “**Transient expression of AP2IX-1 induces surface antigen switching**”, we have performed at least two entirely separate transfection experiments on different days each with three biological replicates (i.e. three independent samples transfected and then measured). The qPCR measurement was performed for four times for each biological replicate. These information can be found in Figure 5 caption and in Materials and Methods section. Criteria for exclusion of single-cell data due to poor sequencing or amplification criteria is described in Materials and Methods, and the preprocessing scripts containing the parameters are available on <https://github.com/xuesoso/singleToxoplasmaSeq>. All of our raw and processed data are available on Gene Expression Omunibus and SRA with the accession number GSE145080, as stated in Materials and Methods section.

- Statistical analysis methods should be described and justified
- Raw data should be presented in figures whenever informative to do so (typically when N per group is less than 10)
- For each experiment, you should identify the statistical tests used, exact values of N, definitions of center, methods of multiple test correction, and dispersion and precision measures (e.g., mean, median, SD, SEM, confidence intervals; and, for the major substantive results, a measure of effect size (e.g., Pearson's r, Cohen's d)
- Report exact p-values wherever possible alongside the summary statistics and 95% confidence intervals. These should be reported for all key questions and not only when the p-value is less than 0.05.

Please outline where this information can be found within the submission (e.g., sections or figure legends), or explain why this information doesn’t apply to your submission:

(For large datasets, or papers with a very large number of statistical tests, you may upload a single table file with tests, Ns, etc., with reference to sections in the manuscript.)

We provided details on the meaning of error bars in the Figure caption. For details on the statistical techniques used, we provided information in both the Results section as well as in Materials and Methods section.

**Group allocation**

- Indicate how samples were allocated into experimental groups (in the case of clinical studies, please specify allocation to treatment method); if randomization was used, please also state if restricted randomization was applied
- Indicate if masking was used during group allocation, data collection and/or data analysis

Please outline where this information can be found within the submission (e.g., sections or figure legends), or explain why this information doesn’t apply to your submission:

**Additional data files (“source data”)**

- We encourage you to upload relevant additional data files, such as numerical data that are represented as a graph in a figure, or as a summary table

Information is available in Figure caption and Materials and Methods section.

- Where provided, these should be in the most useful format, and they can be uploaded as “Source data” files linked to a main figure or table
- Include model definition files including the full list of parameters used
- Include code used for data analysis (e.g., R, MatLab)
- Avoid stating that data files are “available upon request”

Please indicate the figures or tables for which source data files have been provided:

Source data files are provided for Figure 5f and 5g. All other figures can be regenerated using the processed data objects that we have made available at <https://github.com/xuesoso/singleToxoplasmaSeq> and on Gene Expression Omnibus with accession GSE145080.
